# Optimal Timing of Exercise for Enhanced Learning and Memory: Insights From CA1 and CA3 Regions in Traumatic Brain Injury Model in Male Rats

**DOI:** 10.1002/brb3.70354

**Published:** 2025-03-13

**Authors:** Forouzan Rafie, Sedigheh Amiresmaili, Mohammad Amin Rajizadeh, Mohammad Pourranjbar, Elham Jafari, Mohammad Khaksari, Sara Shirazpour, Omid Moradnejad, Amir Hossein Nekouei

**Affiliations:** ^1^ Neuroscience Research Center, Institute of Neuropharmacology Kerman University of Medical Sciences Kerman Iran; ^2^ Division of General Medicine and Geriatrics Department of Medicine Emory University School of Medicine Atlanta Georgia USA; ^3^ Department of Physiology Bam University of Medical Science Bam Iran; ^4^ Physiology Research Center, Institute of Neuropharmacology Kerman University of Medical Sciences Kerman Iran; ^5^ Pathology and Stem Cell Research Center and Department of Pathology Kerman University of Medical Science Kerman Iran; ^6^ Department of Physiology and Pharmacology Kerman Medical Science University Kerman Iran; ^7^ Department of Epidemiology and Biostatistics, School of Public Health Kerman University of Medical Sciences Kerman Iran

**Keywords:** anxiety, brain edema, cognitive, delay exercise, hippocampus, immediate exercise, TBI

## Abstract

**Objective:**

Evidence suggests that exercise timing is crucial in reducing the impact of traumatic brain injury (TBI). The present study explores the effects of delayed and early exercise on brain damage, cognitive dysfunction, and anxiety behavior using an experimental TBI model.

**Methods:**

We randomly assigned 36 male rats to six groups: control (sham, TBI), treadmill exercise (24hA, 1‐month exercise 24 h after TBI), 1WA (1‐month exercise 1 week after TBI), 1MB (1‐month exercise before TBI), and 1MBA (1‐month exercise before and after TBI).

**Results:**

TBI caused significant impairments in cognitive and anxiety behaviors, as well as increased brain edema (*p *< 0.05). The exercise groups showed significant improvement in the following order for cognitive impairments: 1MBA > 24hA > 1WA > 1MB. Compared to the 1WA group, exercise starting 24 h after TBI (24hA) significantly improved all variables except anxiety behavior. Exercise 1MBA was significantly more effective than other groups (*p* < 0.05) in reducing cognitive problems, anxious behavior, and brain damage.

**Conclusion:**

Regular exercise or a consistent exercise routine before TBI, such as in athletes, may provide the most benefits from exercise intervention after the TBI. Starting exercise soon after the TBI (within 24 h) may help protect against brain edema and improve learning and memory by reducing cell death in specific brain regions (CA1 and CA3) and also decreasing TNF‐α and MDA compared to starting exercise later (1 week after).

## Graphical abstract

Early and late exercise can improve cognitive impairments following TBI by reducing inflammation, oxidative stress, and neuronal damage.

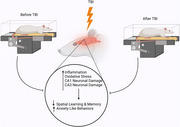

## 1 Introduction

Traumatic brain injury (TBI) is a major cause of injury and death globally, impacting millions of individuals annually. TBI is a diverse condition that can occur due to various reasons, such as falls, car crashes, sports injuries, and assaults. TBI can result in a variety of physical, thinking, and emotional challenges that greatly affect a person's daily life, social interactions, and work abilities (Langlois et al. 2006).

Working memory, executive functions, long‐term memory, and attention are among the cognitive functions that TBI affects (Dehghan et al. 2018; Dewan et al. 2018). Researchers have reported these cognitive impairments in the initial hours and days following trauma (Bland et al. 2011). Moderate to severe TBI significantly hinders the ability to return to work, sometimes lasting up to a year after the injury (Ding et al. 2005). According to Clark et al. (2008), mood problems like depression peak in the first 3 months after the trauma, but signs and symptoms of anxiety and depression can also manifest years later. Research shows that damage to sensory areas, the dorsal parietal cortex, and the frontal area of the brain (responsible for short‐term memory), along with the duration of unconsciousness, can reduce TBI patients' memory performance (Mota et al. 2012). Low memory function is a significant factor in predicting mental disorders after trauma. It can also serve as the basis for the occurrence of such disorders (Fehrenbach and Schneider 2006; Soltani et al. 2016). Even mild TBI (mTBI) can result in serious consequences, including amnesia after trauma (Ding et al. 2005) and debilitating cognitive disorders that range from extensive deficits to severe and chronic psychosis (Taguchi et al. 2019). Despite medication and surgery, some complications of TBI may persist unresolved until the end of the patient's life (Ko et al. 2018).

Exercise has been shown to have neuroprotective and anti‐inflammatory effects. It also improves cognitive function and emotional well‐being in both healthy individuals and those with neurological conditions (Khoramipour et al. 2023; Rafie et al. 2023). Studies have shown that pre‐injury exercise can prevent primary inflammatory and oxidative responses following TBI. Engaging in sports activities before brain trauma can help prevent brain damage and reduce injury size after ischemia (Thornton et al. 2005). Late exercise improved neurological and motor outcomes in rats with TBI by reducing injury size and increasing markers that prevent cell death (Piao et al. 2013). Regular exercise leads to metabolic changes, reduces cell damage and inflammation, and improves post‐disability outcomes from TBI by generating new nerve cells and blood vessels (Silva et al. 2013).

The optimal timing for initiating exercise following TBI remains uncertain. Prior research has demonstrated that early exercise intervention post‐TBI can augment neuroplasticity and enhance functional recovery (Griesbach et al. 2007). Conversely, late exercise intervention has been found to ameliorate cognitive function and diminish neuroinflammation in animal models of TBI (Piao et al. 2013). Consequently, the most opportune timing for exercise intervention post‐TBI remains undetermined.

Studies in rodents (Jacotte‐Simancas et al. 2015; Perry et al. 2020) have shown that training after TBI improves neurological outcomes. The hippocampus, a part of the brain, is important for learning, memory, and controlling emotions. The hippocampus is especially prone to damage after a TBI. Its dysfunction has been linked to the cognitive and emotional difficulties often seen in TBI survivors (Rajizadeh et al. 2023). The hippocampus consists of different parts, like the CA1 and CA3 areas, which are very sensitive to damage from traumatic brain injuries (Amirazodi et al. 2022; Khaksari et al. 2018). Although both the CA1 and CA3 regions contribute to learning and memory, they have distinct functions in forming and recalling memories (Shafiei et al. 2022). Recent research indicates that the CA1 and CA3 regions may respond differently to damage after a TBI (Griesemer and Mautes 2007; Mao et al. 2013).

Hence, studying the effects of exercise on the CA1 and CA3 regions individually is vital to understanding how exercise impacts each region's recovery. Despite significant advances in diagnosis and treatment, TBI remains a major public health problem with limited effective therapeutic options.

Current treatments for TBI are limited and often prioritize symptom management rather than recovery promotion. Medications are available but have limited effectiveness and can lead to side effects. Non‐pharmacological interventions, such as exercise, offer promising ways to manage TBI's physical, cognitive, and emotional outcomes. However, the effects of exercise on damage caused by TBI are a topic of debate, largely dependent on the appropriate timing of exercise initiation following a brain injury. The point of this study is to find the best time for people who have had a TBI to start exercise programs. The study will also look at how these programs affect neuroinflammation, anxiety behavior, and cognitive dysfunction in a model setting. It will also look at the effects on the CA1 and CA3 regions. Ultimately, this research may contribute to developing new therapeutic strategies for TBI based on exercise interventions.

## 2 Materials and Methods

### 2.1 Animals

Thirty‐six adult male Wistar rats weighing 250–300 g were kept in a room with a constant temperature of 22 ± 2°C and a 12‐h light/12‐h dark cycle. Animals had access to water and food. This study's research adhered to global animal welfare standards. The ethics committee at Kerman University of Medical Sciences (No. IR.KMU. REC.1398.245) approved it and conducted it according to the ARRIVE guidelines.

We divided the animals into six groups, each containing six rats. The NE groups consisted of the sham and TBI NE groups. The TBI exercise groups, included early initiation group (24hA): exercise started 24 h after TBI and continued for 4 weeks, late initiation group (1WA): exercise began 1 week after TBI and lasted 4 weeks, prophylactic group (1MB): exercise started 4 weeks before TBI, prophylactic and therapy group (1MBA): exercise started 4 weeks before TBI and continued for 4 weeks after TBI (Rafie et al. 2022).

### 2.2 Exercise Regimen

The rats in the exercise groups were introduced to the treadmill by using it for 10 min daily for 5 days without running. Following this, as part of the primary exercise routine, the rats ran on the treadmill for 30 min once a day, 5 days a week, over 4 weeks. The exercise regimen included running at a zero‐degree incline, commencing at a speed of 2 m/min for 5 min, progressing to 5 m/min for the subsequent 5 min, and maintaining 8 m/min for the remaining 20 min. In contrast, the control group rats were placed on the treadmill without running for the same duration as the exercise groups. Cognitive and behavioral assessments were conducted 24 h after the final exercise session (Rafie et al. 2022).

### 2.3 Induction of mTBI

In our study, we employed the Marmarou impact acceleration model, a validated technique extensively used to simulate diffuse TBI, particularly mTBI, in a controlled and replicable manner. This model effectively replicates the diffuse axonal injury observed in clinical mTBI cases, such as falls or car accidents. To induce an mTBI in the animals, we administered ketamine and xylene anesthesia. A 10 mm‐diameter and 3 mm‐thick steel plate was affixed to the skull using polyacrylamide glue at the position between the bregma and lambda sutures (Farahani et al. 2022). An incision was made in the scalp to expose the skull before attaching the plate. Keshavarzi et al. (2021) detailed the plate placement between the bregma and lambda sutures along the coronal suture. For consistent diffuse axonal injury induction, we utilized a 300 g weight drop from a height of 2 m onto the steel plate. This technique, applied to animals positioned on foam for impact absorption, is well‐supported in literature for reliably producing mTBI markers like axonal damage and neuroinflammation (Amirkhosravi et al. 2021). Prior studies using the same parameters (2 m height, 300 g weight) have shown consistent neuroinflammatory and axonal injury responses, aligning well with clinical mTBI presentations (Akçay 2023; Dehghan et al. 2018).

### 2.4 Morris Water Maze Test

The Morris water maze test, conducted in a single day, assessed the animals' learning and spatial memory abilities. The pool was geographically divided into four quadrants of equal size, and starting points were designated at each quadrant as north (N), south (S), east (E), and west (W). A square platform (10 cm in diameter) was hidden right below (1.5 cm) the surface of water in the center of the northeast quadrant. The test comprised three blocks, each with a 30‐min interval. In each block, the animal was released randomly from one of the four quadrants into the water, with a maximum of 60 s to locate a hidden platform. The experiments were carried out in a dimly lit room with various fixed extra maze geometric images (e.g., circles, squares, or triangles) attached to different points on the walls around the maze. After finding the platform, the animal rested for 30–35 s before returning to its original position under a lamp. Learning was evaluated based on performance metrics such as the distance traveled to reach the platform, the time taken to find the platform, and the swimming speed of the animals. Consistency was maintained in subsequent Morris water maze test trials, with the animals being released from different quadrants following the same protocol. The probe test to assess spatial memory was conducted 2 h after the final learning trial. During the probe test, the hidden platform was removed, and the animal was released into the water from the opposite quadrant of the target circle and observed for 60 s of free‐swimming behavior (Esmaeilpour et al. 2024; Khajei et al. 2021).

### 2.5 Open Field

We used the open‐field (OF) test to evaluate anxiety and exploration tendencies. This test involves placing the animal in a Plexiglas box that measures 80 cm × 80 cm × 50 cm with controlled lighting, temperature, and noise levels. The rat is positioned in the middle of the box, and an overhead camera continuously records its movements for 5 min. To measure grooming behavior and rearing in the OF box, we analyzed the recorded motion data using a Noldus EthoVision system (v7.1, the Netherlands) (Shahraki et al. 2022).

### 2.6 Evaluation of Neurological Outcomes

Neurological outcomes were assessed using the VCS scoring system, which includes motor situation (ranging from 1 to 8, with higher scores indicating better motor function), eye situation (ranging from 1 to 4, with higher scores indicating better eye function), and respiration situation (ranging from 1 to 3, with higher scores indicating better respiration function). The VCS score ranges from 3 to 15, where higher scores indicate better neurological outcomes and lower scores indicate worse ones. The VCS was evaluated 1 h before surgery and at 1, 4, and 24 h after surgery, as previously described by Soltani et al. (2022).

### 2.7 Sampling

After performing behavioral tests, all rats were sacrificed with lethal doses of ketamine (100 mg/kg) and xylazine (50 mg/kg). The animals were decapitated, and the brain samples were taken from rats. One hemisphere was placed in the oven to check the water content of the brain. From the other hemisphere, the hippocampus was separated, and a part of it was placed in 10% formalin for histological studies, and the other part was frozen and kept for molecular evaluations.

### 2.8 Evaluation of the Brain Edema

Six brain samples in each group were utilized for brain edema evaluation. The brain tissue was kept at 60°C for 24 h to measure the amount of dry tissue weight, and the brain water content (BWC) was determined through the following formula: BWC (%) = (100 × [(wet weight − dry weight)/wet weight]) (Soltani et al. 2020).

### 2.9 Histopathological Analysis

The hippocampus of the brains was removed, fixed in 10% buffered formaldehyde, sliced into 5 µm sections using an automatic microtome (LEICA, Germany), and stained with hematoxylin–eosin. Two pathologists blinded to the animal group examined the CA1 and CA3 apoptosis using a microscope with a 10× magnification (Olympus, CA 33, Japan). Apoptosis was scored based on its severity, which was graded on a scale of (0) scant, (1) mild, (2) moderate, and (3) severe. Scant apoptosis was observed when it was hardly visible in a high‐power field; mild apoptosis was observed when it was visible in a high‐power field; moderate apoptosis was observed when it was visible in more than one field; and severe apoptosis was observed when it was visible in almost all of the high‐power fields. Brain edema was evaluated by observing spaces in the extracellular spaces that separated parenchymal cells and was graded as mild (when edema was < 50%) or severe (when edema was ≥ 50%) using a microscope with a magnification of 10× (Rafie et al. 2022).

### 2.10 Biochemical Measurements in Hippocampus Tissue

The Bradford method was used to determine the total protein in hippocampus tissue. Malondialdehyde (MDA), as an index of lipid peroxidation, was estimated using the concentration of thiobarbituric acid reactive substances (TBARS) at 550 nm according to the kit's instructions (Navand Salamat Co., Iran). Total antioxidant capacity (TAC) was determined by the ferric‐reducing ability of plasma (FRAP) assay at 593 nm via the related kit (Navand Salamat Co., Iran). Quantitative assessments of hippocampus TNF‐α were conducted using the double‐antibody sandwich enzyme‐linked immunosorbent assay (ELISA) kits based on the manufacturers’ instructions (Karmania Pars Gene Co., Iran).

### 2.11 Statistical Analysis

The statistical analysis used GraphPad Prism 6.00 for Windows (GraphPad Software, La Jolla, California, USA). The normal distribution of all the data was assessed using the Shapiro–Wilk test. Descriptive analysis was performed using mean and standard error values. The one‐way analysis of variance (ANOVA) method was used to examine the results, with the Tukey's test used for post hoc analysis. The data was expressed as mean ± SEM, and statistical significance was determined at a *p* value of less than 0.05.

## 3 Results

### 3.1 Exercise Prevented the Development of Brain Edema Following TBI

**FIGURE 1:**
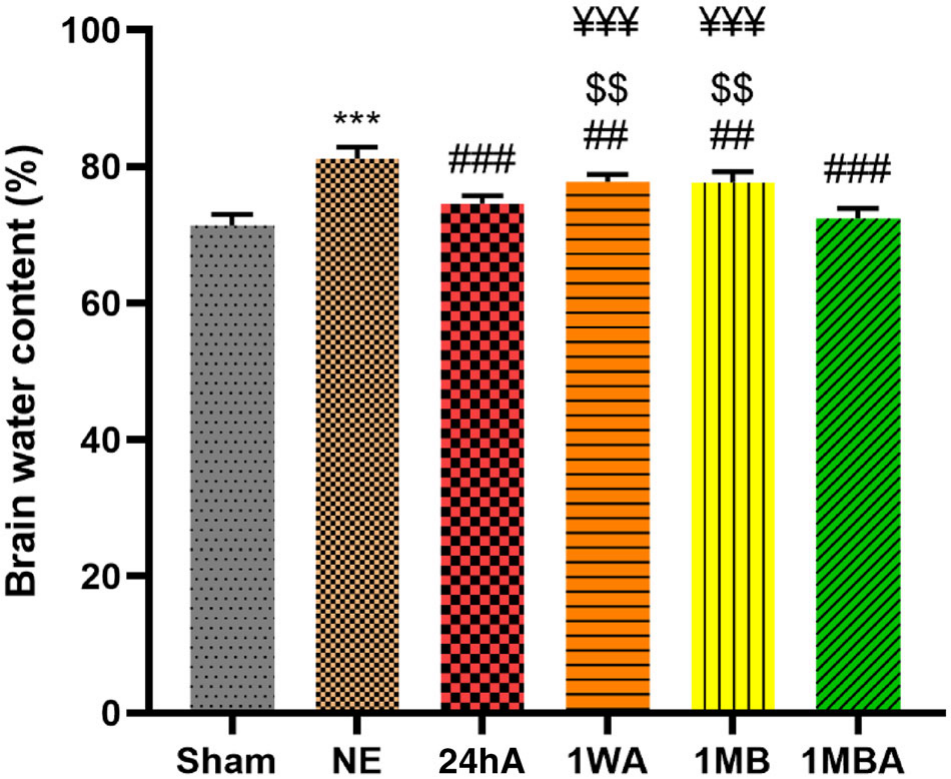
The results are represented as mean ± SEM. (*n* = 7). ***p < 0.001, versus sham. ^###^ *p* < 0.001 and **^##^** *p *< 0.01 versus NE. ^$$^ *p* < 0.01 versus 24 h. ^¥¥¥^ *p* < 0.001 versus 1MBA. 1MB: 1 month before TBI, 1MBA: 1 month before and after TBI, 1WA: 1 week after TBI, 24hA: 24 h after TBI, TBINE: TBI no exercise.

The effect of exercise on brain edema determined via BWC 24 h post‐TBI is shown in Figure 1. One‐way ANOVA showed that there was a significant difference among groups (*F*(5, 30) = 40, *p *< 0.001). Tukey's test showed that the TBI group had a higher BWC than the sham group (*p *< 0.001). The exercise prevented the increase in BWC following TBI in comparison with the TBI group (*p *< 0.001 for 24hA and 1MBA and *p *< 0.01 for 1WA and 1MB).

### 3.2 Neurological Outcome Improved Following TBI

**FIGURE 2:**
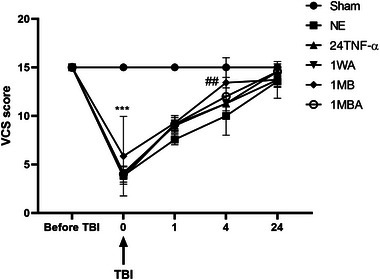
Veterinary coma scale (VCS) in exercised male rats at –1, 1, 4, and 24 h post‐TBI (*n *= 6 in each group). Data are shown as mean ± SEM. ***p < 0.001 compared to the sham group. ^##^ *p* < 0.01 compared to NE.

In rates with the history of exercise, the alteration in the neurological outcome by evaluating VCS at −1, 1, 4, and 24 h post‐TBI in the presence of previous treadmill training is shown in Figure 2. Before the trauma, the groups had no statistical difference in VCS. A decrease in VCS appeared in the TBI group compared to the sham and exercise sham groups at the evaluation times post‐TBI.

### 3.3 Spatial Learning

**FIGURE 3:**
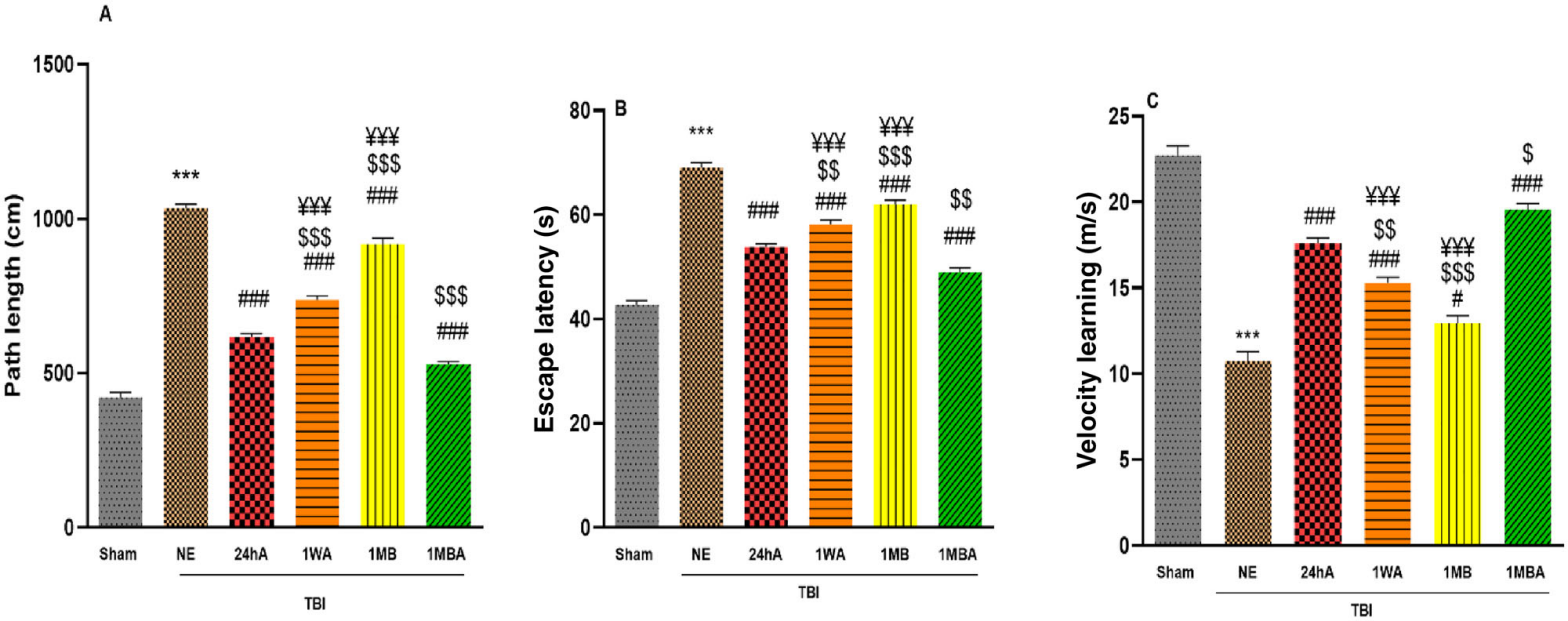
Effects of the different initiation times of exercise on (A) distance, (B) escape latency, and (C) velocity in learning (each group *n* = 7). The results are represented as mean ± SEM. ***p < 0.001 versus sham. ^###^ *p* < 0.001 and ^#^ *p *< 0.05 versus NE. ^$^ *p *< 0.05 and ^$$^ *p* < 0.01 and ^$$$^ *p *< 0.001 versus 24h. ^¥¥¥^ *p* < 0.001 versus 1MBA. 1MB: 1 month before TBI, 1MBA: 1 month before and after TBI, 1WA: 1 week after TBI, 24hA: 24 h after TBI, TBINE: TBI no exercise.

Two‐way ANOVA with repeated measurement tests showed that there was a significant difference among groups (*F*(5, 30) = 280.2, *p *< 0.001). The path length in the sham group was lower than in the NE group (*p *< 0.001). The distance traveled to reach the platform in the TBI exercise groups was also lower than in the NE group (*p *< 0.001). In addition, the amount of this index in the 1MBA group decreased compared to the 24hA, 1WA, and 1MB groups (*p *< 0.01). This indicator in the 24hA group was lower than in the 1WA and 1MB groups (Figure 3A).

Two‐way ANOVA with repeated measurement tests showed that there was a significant difference among groups (*F*(5, 30) = 134.5, *p *< 0.001). The analysis of the escape latency to find a platform in all groups showed a significant difference between groups. The mean elapsed time to reach the platform in the NE group was higher than that in the sham group (*p *< 0.001). This index in the NE group was higher than in other exercised groups (*p *< 0.001). The mean escape latency to find the platform following 1MBA was lower than 24hA, 1WA, and 1MBA groups (*p *< 0.01). Moreover, this indicator in the 24hA group was lower than in the 1WA and 1MB groups; there is a significant difference between the 1MB and 1WA groups (*p *< 0.001) (Figure 3B).

Two‐way ANOVA with repeated measurement tests showed that there was a significant difference among groups (*F*(5, 30) = 103.1, *p *< 0.001). The velocity (m/s) analysis in all groups showed a significant difference. The velocity in all TBI groups was lower than in the sham group (*p *< 0.001). Exercised rats learned to find the platform quickly, so the velocity in the 1MBA rats was greater than in the TBI, 1MB, 1WA, and 24hA groups. This index in the 24hA group was higher than in the 1WA and 1MB groups (*p *< 0.001) (Figure 3C).

### 3.4 Spatial Memory

**FIGURE 4:**
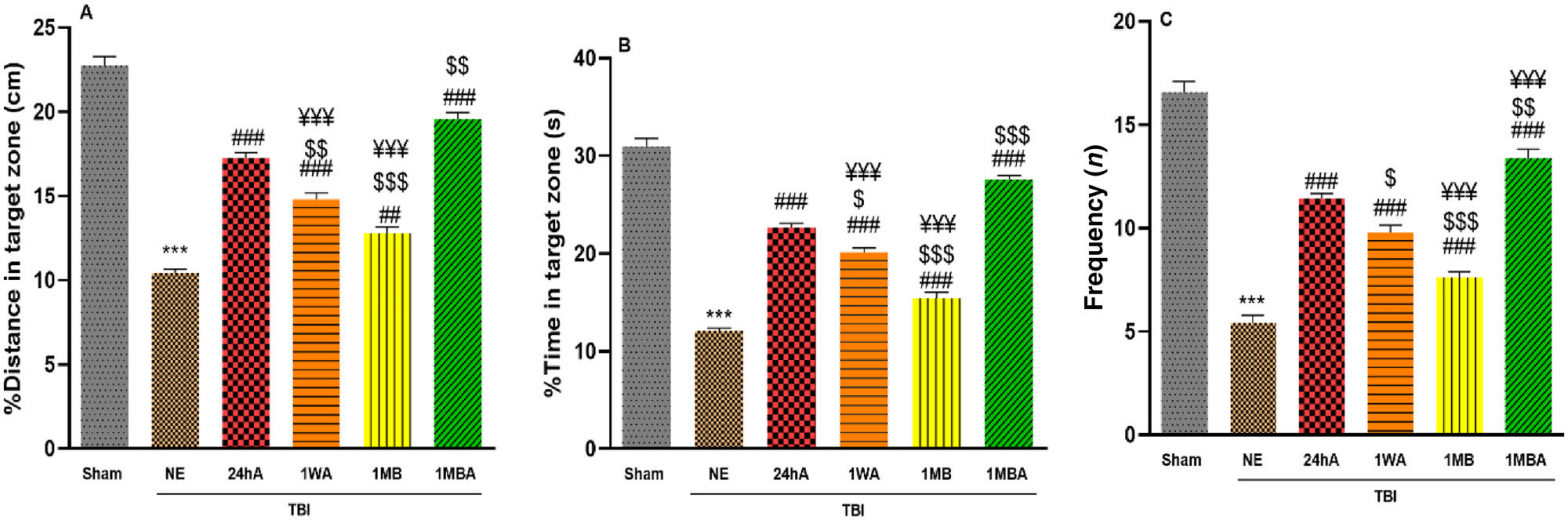
Effects of the different initiation times of exercise on (A) %distance in the target zone, (B) % time in the target zone, and (C) frequency in memory (each group *n* = 7). The results are represented as mean ± SEM. ***p < 0.001 versus sham. ^###^ *p* < 0.001 and ^##^ *p *< 0.01 versus NE. ^$^ *p *< 0.05 and ^$$^ *p* < 0.01 and ^$$$^ *p *< 0.001 versus 24h. ^¥¥¥^ *p* < 0.001 versus 1MBA. 1MB: 1 month before TBI, 1MBA: 1 month before and after TBI, 1WA: 1 week after TBI, 24hA: 24 h after TBI, TBINE: TBI no exercise.

2 h later, the memory probe test was performed by removing the hidden platform. Figure 4A,B illustrate the percentage of time and distance spent in the target quadrant during the memory phase in the study groups.

One‐way ANOVA showed that there was a significant difference among groups (*F*(5, 30) = 136, *p *< 0.001). The distance percentage moved in the target quadrant significantly decreased in NE compared with the sham group (*p *< 0.001). Our results indicated that the distance rate in the target quadrant following the 1MBA exercise was increased compared to the 24hA, 1WA, and 1MBA exercise groups, and this index in the 24hA group was higher than that in the 1WA and 1MB groups (*p *< 0.01). A significant difference was also observed between the 1MB and 1WA groups (*p *< 0.001).

One‐way ANOVA showed that there was a significant difference among groups (*F*(5, 30) = 184.3, *p *< 0.001). The time percentage spent in the target quadrant significantly decreased in NE compared with the sham group (*p *< 0.001). Exercised rats spent more time in the target zone, so the time percentage in the 1MBA rats was higher than in NE and other exercise groups (*p *< 0.001). This index in the 24hA group was higher than the 1WA and 1MB groups (*p *< 0.001).

One‐way ANOVA showed that there was a significant difference among groups (*F*(5, 30) = 114.5, *p *< 0.001). The results of the frequency arrival to the target quadrant in the probe test are shown in Figure 4C. The number of arrivals to the target quadrant was lower in the TBI groups than in the sham group (*p *< 0.001). In addition, this number was reduced in the NE group compared to the exercise groups (*p *< 0.001). In contrast, an increase in frequency was observed in the 1MBA exercise group compared to other exercise groups (*p *< 0.001); besides, this index in the 24hA group was higher than that in the 1MB and 1WA groups (*p *< 0.01 and *p *< 0.001). A significant difference was also observed between the 1MB and 1WA groups (*p *< 0.001) (Figure 4C).

### 3.5 Anxiety‐Like Behavior

#### 3.5.1 Rearing

**FIGURE 5:**
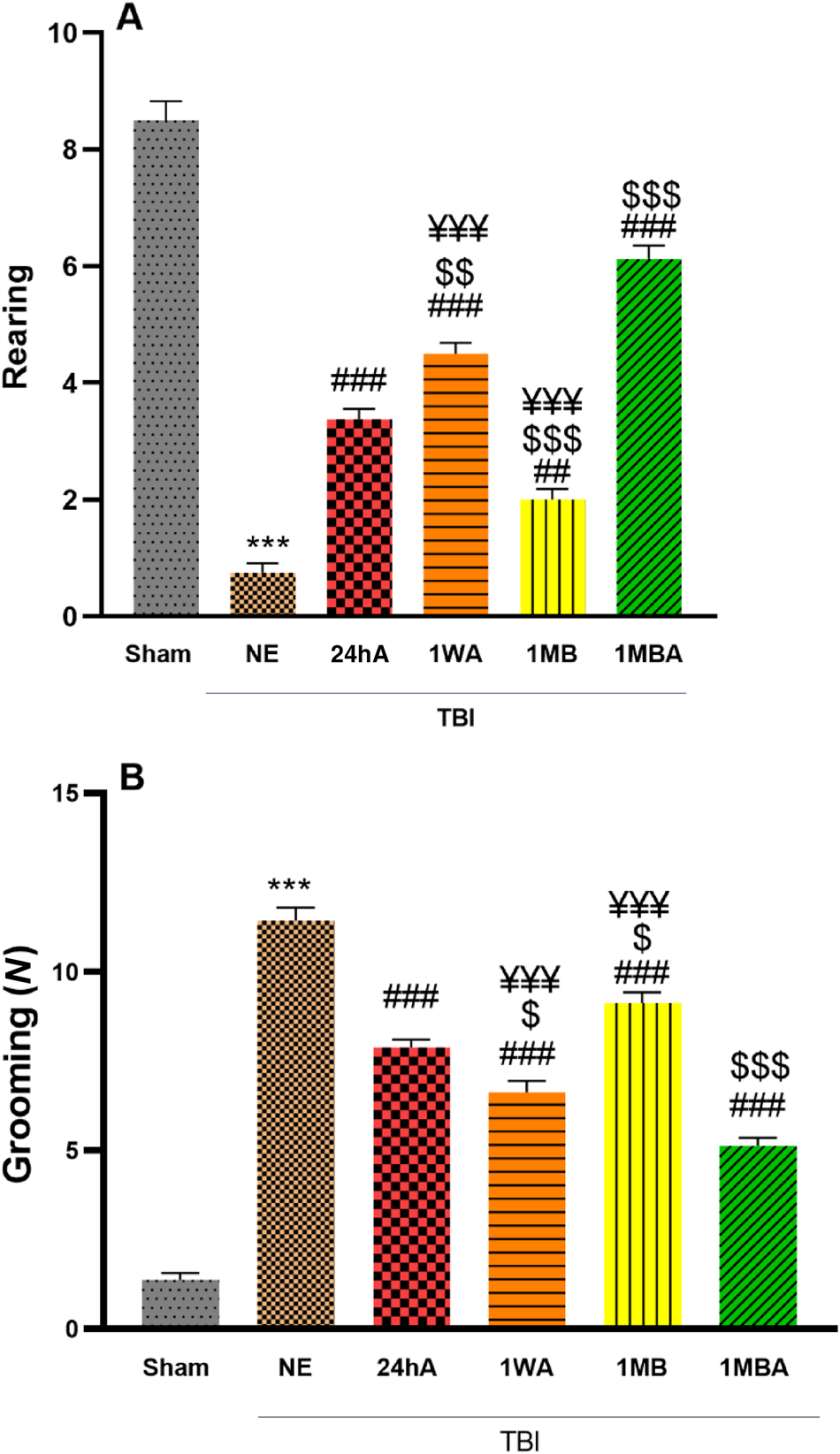
Effects of the different initiation times of exercise on (A) rearing and (B) grooming in all groups (each group *n* = 7). The results are represented as mean ± SEM. ***p < 0.001 versus sham. ^###^ *p* < 0.001 and ^##^ *p *< 0.01 versus NE. ^$^ *p *< 0.05 and ^$$^ *p* < 0.01 and ^$$$^ *p *< 0.001 versus 24h. ^¥¥¥^ *p* < 0.001 versus 1MBA. 1MB: 1 month before TBI, 1MBA: 1 month before and after TBI, 1WA: 1 week after TBI, 24hA: 24 h after TBI, TBINE: TBI no exercise.

This study assessed anxiety‐like behaviors by measuring the number of rearing and grooming in the OF. The analysis of the rearing showed a significant difference between groups (*F*(5, 30) = 110.6, *p *< 0.001). TBI rats performed less rearing compared with the sham group (*p *< 0.001). In addition, this index in the NE group was lower than in the exercise groups (*p *< 0.001). The exercise reduced the anxiety behavior in the 1MBA and 24hA groups with a significant increment in rearing compared to other exercise groups (*p *< 0.001) (Figure 5A).

#### 3.5.2 Grooming

Figure 5B illustrates a significant difference in grooming between groups (*F*(5, 30) = 114.1, *p *< 0.001). The grooming significantly increased in the TBI groups compared with the sham group (*p *< 0.001). Following exercise, the grooming significantly decreased, so in the TBI exercise group, grooming was lower than in the TBI NE group (*p *< 0.001). The 1MBA group had the lowest grooming after TBI among all the exercise groups (*p *< 0.001). Also, the 24hA group showed a more significant reduction in grooming than the 1WA and 1MB groups (*p *< 0.001) (Figure 5B).

#### 3.5.3 Inner Zone and Outer Zone Duration

The analysis of the inner zone duration (*F*(5, 30) = 76.14, *p* < 0.001) and outer zone duration (*F*(5, 30) = 110.6, *p *< 0.001) showed a significant difference between groups. Our results showed that TBI increased time spent in the periphery of the box or outer zone duration while reducing time spent in the center or inner zone duration compared to the sham group (*p *< 0.001). On the other hand, the inner zone duration in exercise groups was higher than the TBI group, while the outer zone duration was lower (*p *< 0.001).

### 3.6 Histopathological Consideration

**FIGURE 6:**
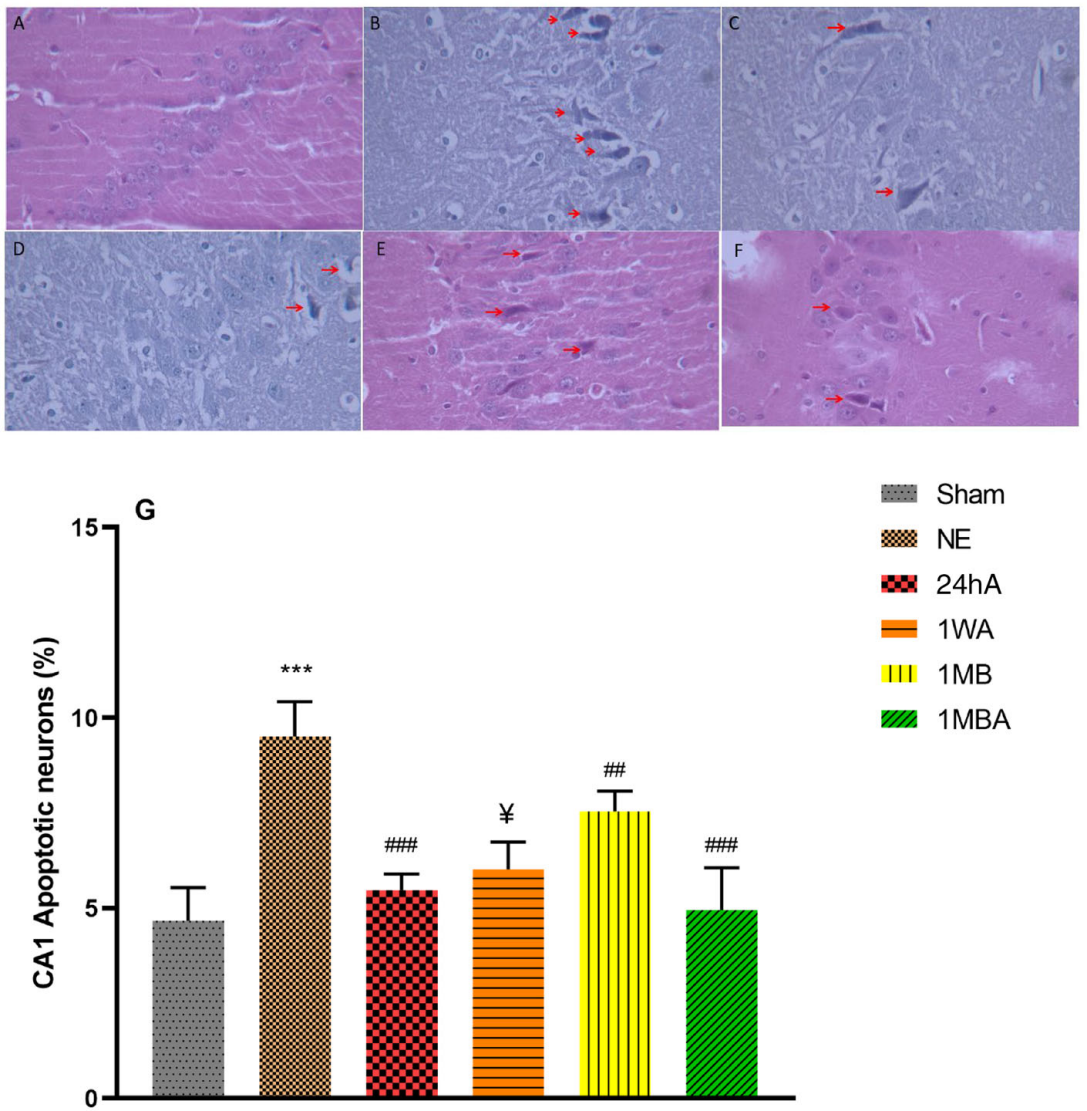
Effects of the different initiation times of exercise on histopathological changes in different groups in the CA1 area of the hippocampus (magnification 400×). Red arrows indicate apoptosis in the CA1 area of the hippocampus. (G) Apoptotic neurons percentage in all groups (each group *n* = 8). (A) Sham, (B) NE: no exercise, (C) 24hA: 24 h after TBI, (D) 1WA: 1 week after TBI, (E) 1MB: 1 month before TBI, (F) 1MBA: 1 month before and after TBI. The results are represented as mean ± SEM. ***p < 0.001 versus sham. ^###^ *p* < 0.001 and ^##^ *p *< 0.01 versus NE. ^$$$^ *p *< 0.001 versus 24h. ^¥¥¥^ *p* < 0.001 versus 1MBA.

**FIGURE 7:**
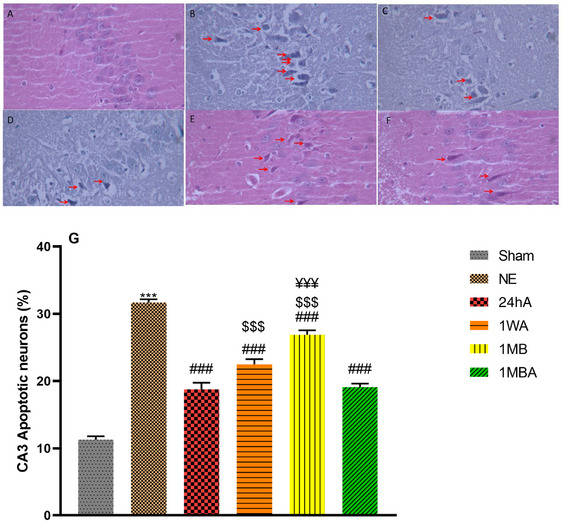
Effects of the different initiation times of exercise on histopathological changes in different groups in the CA3 area of the hippocampus (magnification 400×). Red arrows indicate apoptosis in the CA3 area of the hippocampus. (G) Apoptotic neurons percentage in all groups (each group *n* = 8). (A) Sham, (B) NE: no exercise, (C) 24hA: 24 h after TBI, (D) 1WA: 1 week after TBI, (E) 1MB: 1 month before TBI, (F) 1MBA: 1 month before and after TBI. The results are represented as mean ± SEM. ***p < 0.001 versus sham. ^###^ *p* < 0.001 versus NE. ^$$$^ *p *< 0.001 versus 24h. ^¥¥¥^ *p* < 0.001 versus 1MBA.

The analysis of the CA1 neurons (*F*(5, 30) = 32.06, *p *< 0.001) and CA3 neurons (*F*(5, 30) = 99.78, *p *< 0.001) showed a significant difference between groups. The effect of TBI and exercise on neuronal apoptosis was assessed through histopathological evaluation of hippocampal CA1 (Figure 6A–G) and CA3 (Figure 7A–G) pyramidal neurons. The histopathological evaluation shows that the percentage of apoptotic cells in the TBI groups was higher than in the sham group (*p *< 0.001). Although these indices in the NE group were higher than in other exercise TBI groups, the apoptotic cells in 1MBA were lower than in other exercise groups. In addition, the indices in the 24hA were lower than 1WA and 1MB (*p *< 0.001).

### 3.7 Inflammation and Oxidative Stress

**FIGURE 8:**
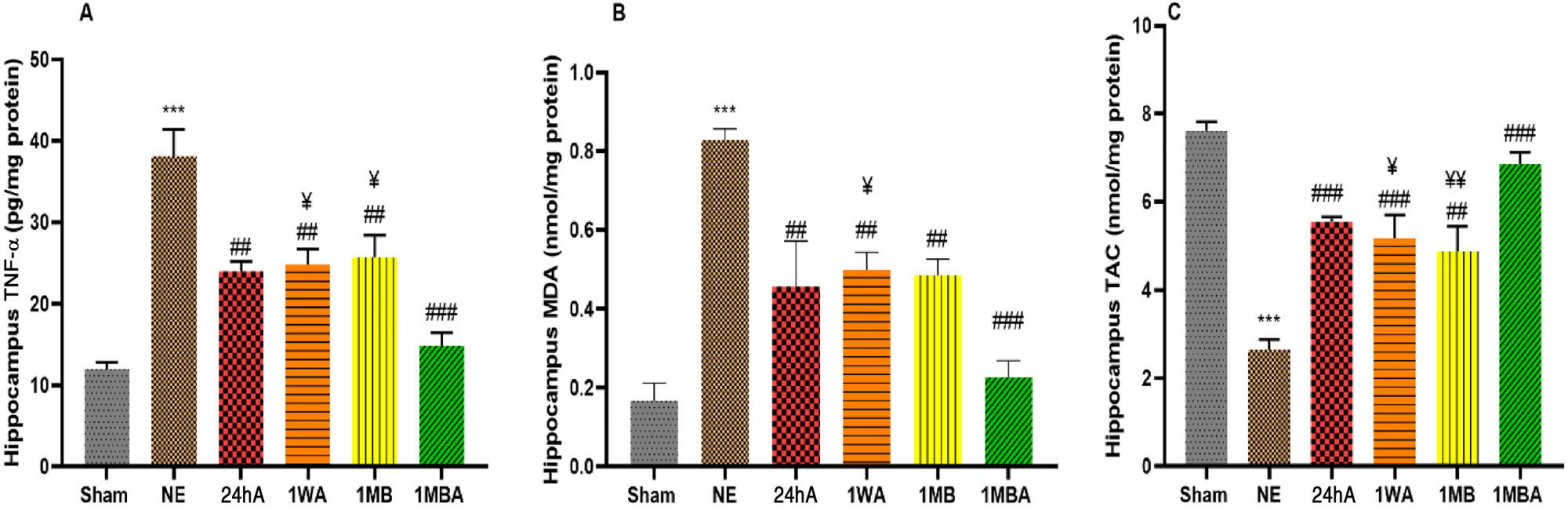
Effects of the different initiation times of exercise on (A) hippocampus tissue TNF‐α, (B) hippocampus tissue MDA, and (C) hippocampus tissue TAC in all groups (each group *n* = 7). The results are represented as mean ± SEM. ***p < 0.001 versus sham. ^###^ *p* < 0.001 and ^##^ *p *< 0.01 versus NE. ^¥¥^ *p* < 0.01 and ^¥^ *p *< 0.05 versus 1MBA. TBINE: TBI no exercise, 24hA: 24 h after TBI, 1WA: 1 week after TBI, 1MB: 1 month before TBI, 1MBA: 1 month before and after TBI.

One‐way ANOVA showed that there was a significant difference among groups regarding TNF‐α (*F*(5, 30) = 13.75, *p *< 0.001), MDA (*F*(5, 30) = 13.90, *p *< 0.001), and TAC (*F*(5, 30) = 17.10, *p *< 0.001). In the NE group, the levels of TNF‐α and MDA in hippocampus tissue were significantly higher than in the sham group (*p *< 0.001), while the TAC level was lower than in the sham group (*p *< 0.001). Exercise improved inflammation and oxidative stress by reducing TNF α and MDA and increasing TAC levels in the hippocampus (*p *< 0.001) (Figure 8).

## 4 Discussion

Individuals with TBI often face challenges with motor skills and anxiety, which affect their daily lives, social interactions, and well‐being (Born et al. 2018). Exercise is recognized as a beneficial and cost‐effective way to address TBI‐related issues (Perry et al. 2020). However, conflicting findings and varying recommendations exist regarding the best time to exercise. Studying the specific effects of physical activity on distinct brain areas can offer valuable insights into TBI recovery mechanisms. Targeting neuroinflammation, anxiety, and cognitive issues with customized exercises at key points may improve outcomes for TBI patients. This study investigates how the timing of exercise initiation affects cognitive and behavioral impairments in a TBI rat model.

Our research has found that TBI leads to an increase in cerebral edema. This is consistent with other studies that have taken a similar approach to trauma, which have also shown that this method leads to an increase in cerebral edema (Khaksari et al. 2013; Khaksari et al. 2018). Several mechanisms may be responsible for the increase in BWC, including an increase in inflammatory factors and free radicals, mitochondrial dysfunction, reduced antioxidant enzymes, lipid peroxidation, disruption of the blood‐brain barrier, and loss of neurons (Morganti‐Kossmann et al. 2001; Tuttolomondo et al. 2014). Our study found that the groups treated with exercise showed a decrease in BWC (an indicator of brain edema) compared to the groups with TBI but no exercise.

Groups 1MBA and 24hA decreased brain edema more than the other groups. Our findings are in agreement with the study by Nishioka et al. (2016), which showed that early exercise (after 24 h) improved brain edema in brain injury. Additionally, the study by Park et al. (2010) reported that early treadmill training did not increase hematoma size or BWC after hemorrhagic stroke in rats. Luo et al. (2007) also found that lesion size was reduced, and the number of NeuN‐positive cells was increased in the early exercise group. Finally, the study by Chen et al. (2013) found that early exercise in animals with closed head injury prevented the loss of neurons and neuroinflammation in comparison to late exercise. Research has shown that exercise before experiencing a stroke can help reduce brain damage, cerebral edema, and neurological deficits. This may be due to the stimulation of specific signal pathways, including PKC‐α‐GLT‐1‐s and PI3K/Akt‐GLT‐1‐Glutamate (Wang et al. 2014).

It seems that pre‐exercise increases the tolerance of neurons in injury conditions (Nishioka et al. 2016), and much evidence showed that pre‐rehabilitation effects of exercise are associated with upregulation of the BDNF, NGF, and increased regional angiogenesis (J. Zhang et al. 2012); however, in the Piao study, late exercise initiation beginning at 5 weeks after trauma reduces neuroinflammation after TBI (Piao et al. 2013). These differences, considered with our results, may contribute to optimizing the time of initiating exercise and the kind of exercise (voluntary vs. treadmill running) based on the type of brain injury.

Our results revealed that spatial learning and memory were impaired following TBI. Many investigations showed spatial learning and memory impairment following TBI (Chen et al. 2013; Tarudji et al. 2021). We extend these prior findings to show that exercise can improve impairments following TBI (Ko et al. 2018). Because spatial learning and memory are hippocampus‐dependent, hippocampal damage following TBI can impair these capacities. We show that exercise can improve cognitive impairments by improving hippocampal lesions.

The results of this study show that rats with TBI exhibited increased anxious behaviors, which is consistent with previous research on animals and humans (Archer 2012; Piao et al. 2013). Human and animal studies have shown that staying next to the walls of the maze or thigmotaxis indicates high anxiety (Gromer et al. 2021; Seibenhener and Wooten 2015). The behavior is characterized by the preference of a rodent to seek shelter instead of exposing itself to the aversive open area; that is, a stressed animal is more likely to stay in proximity of the walls and avoid the relatively exposed center of the open area and is less likely to explore the open area/novel environment than a less stressed animal (X. Y. Zhang et al. 2023). Considering the exercise, TBI groups showed the maximal efficacy in 1MBA and delay training groups (1 week later); the physical exercise intervention was recommended in improving anxiety (Ströhle 2009), depression (Fann et al. 2015), sleep problems, and quality of life in patients with TBI (Hoffman et al. 2010). Mychasiuk et al. (2016) compared the effects of early (1–3 days) and delayed (7 days) initiation of exercise post‐concussion and found that delayed initiation of exercise within 7 days resulted in the rehabilitation of anxiety‐like behavior in male rats. Another study demonstrated that late exercise reduced anxiety and increased GABAergic inhibition post‐injury, which could be due to GABA's role in psychiatric disorders such as anxiety and depression in humans (Pietrelli et al. 2012). Yoon and Kim (2018) found a positive impact of immediate low‐intensity exercise on improving behavioral problems. Since stress responses increase following TBI, especially in the first few days (Griesbach et al. 2011), and treadmill running could potentially induce stress, these factors may hinder the positive effects of early exercise (Moraska et al. 2000). Additionally, the differences observed between these studies and our results could be related to the type or severity of the injury (Mychasiuk et al. 2016), animal models (Griesbach et al. 2011), or the duration and intensity of exercise (Shen et al. 2013).

Our study's histopathological results revealed that TBI increased brain apoptosis, inflammation, and bleeding. However, pre‐ and post‐exercise, as well as immediate initiation of exercise, helped to decrease these indicators. These findings are supported by other studies that have shown exercising before CCI (controlled cortical impact) can lead to improved neurological and motor outcomes, as well as reduced apoptosis markers (Acosta et al. 2014; Osier et al. 2015). Physical activity improves neurological dysfunction later by inhibiting apoptosis, weakening neuroinflammation, and neurological repair (Archer 2012; Piao et al. 2013). Also, Ding et al. (2005) reported that treadmill exercise 3 weeks before stroke reduced stroke damage by increasing microvessel density. Recently, one research revealed that pre‐TBI physical exercise reduces the necessary onset delay of post‐TBI exercise to obtain cognitive benefits via reduction of neuronal death, microglial activation, and neuroinflammation in the hippocampus (Sánchez‐Martín et al. 2024). The differences between this study and our study were the model and severity of TBI induction and the type of exercise.

Our observations showed that the levels of TNF‐α increased in the hippocampus following TBI. Consistent with our results, some studies found an increase in TNF‐α levels after brain injury (Khaksari et al. 2015; Rajizadeh et al. 2023). Also, our previous study showed the TNF‐α elevation in serum and CSF of rats after TBI (Rafie et al. 2022). TNF‐α levels in the 24hA group were lower than in the 1WA and 1MB groups. Also, a significant reduction was observed in the 1MBA group compared to other groups. There were no significant differences between 1WA and 1MB in the levels of this cytokine. The adverse effects of increasing the concentration of TNF‐α in the acute phase during recovery have been shown. (Michopoulos et al. 2020), so the protective effects of exercise on this cytokine may inhibit the harmful effects of TNF‐α on the BBB, brain edema, and apoptosis (Dornbos and Ding 2012). Excessive calcium influx into neuronal cells occurs following TBI, leading to the generation of oxidative stress and resulting in mitochondrial dysfunction, lipid peroxidation, and oxidation of proteins and DNA (Emmez et al. 2022). Our data revealed that hippocampal levels of MDA increased, whereas the levels of TAC decreased after brain injury. Other researchers reported that oxidative stress increased following TBI (Petronilho et al. 2010; Tyurin et al. 2000). Our previous study showed that exercise could decrease MDA and increase TAC in serum and CSF (Rafie et al. 2022). Consistent with our results, many investigations showed the protective effects of exercise on oxidative stress following TBI (Sabet et al. 2022; Soltani et al. 2020).

## 5 Conclusion

The study indicates that exercise may significantly improve neurological outcomes for individuals recovering from TBI. This improvement is due to reduced brain edema and optic cell damage, enhanced cognitive and behavioral functions, and lower anxiety levels in those following exercise regimens. Prompt implementation of exercise post‐TBI, especially for individuals with prior regular exercise habits like athletes, could lead to substantial recovery enhancements. Healthcare professionals are advised to consider early exercise rehabilitation for TBI patients.

Our study found that starting exercise within 24 h post‐TBI resulted in more significant improvements in cognitive and motor skills than delayed exercise. This could be attributed to induced neuroprotection, which contributes to better neurological outcomes by reducing edema, apoptotic cell damage, and levels of the inflammatory markers TNF‐α and MDA as an oxidative stress parameter.

However, the impact of exercise on TBI‐induced damage is still a topic of debate, influenced by the severity of the injury. Therefore, we suggest that future research compare this protocol across different TBI severities, including mild and severe cases, and various types of exercises, considering exercise intensity. Additionally, clinical trials are needed to assess the effectiveness of this approach.

## Author Contributions

**Forouzan Rafie**: conceptualization, supervision, writing – original draft, project administration. **Sedigheh Amiresmaili**: methodology. **Mohammad Amin Rajizadeh**: writing – original draft. **Mohammad Pourranjbar**: investigation, validation. **Elham Jafari**: methodology, data curation. **Mohammad Khaksari**: writing – review and editing, project administration, visualization. **Sara Shirazpour**: methodology, data curation. **Omid Moradnejad**: data curation, writing – review and editing. **Amir Hossein Nekouei**: formal analysis, data curation.

## Ethics Statement

The research conducted in this study followed globally accepted standards for animal use and welfare and was approved by the ethics committee (No. IR.KMU. REC.1398.245) at Kerman University of Medical Sciences.

## Consent

The authors have nothing to report.

## Conflicts of Interest

The authors declare no conflicts of interest.

### Peer Review

The peer review history for this article is available at https://publons.com/publon/10.1002/brb3.70354.

## Data Availability

The data are available on rationale request from the corresponding authors.
