# Corrigendum to “Optimal Timing of Exercise for Enhanced Learning and Memory: Insights from CA1 and CA3 Regions in Traumatic Brain Injury Model in Male Rats”

**DOI:** 10.1002/brb3.71795

**Published:** 2026-10-08

F. Rafie, S. Amiresmaili, M. A. Rajizadeh, M. Pourranjbar, E. Jafari, M. Khaksari, S. Shirazpour, O. Moradnejad and A. H. Nekouei, Brain and Behavior 15, no. 3 (2025): e70354, https://doi.org/10.1002/brb3.70354.

The originally published article contained two errors that require correction.

First, the sample size reported in the captions of Figures 1, 3, 4, 5, 6G, 7G, and 8 was incorrectly stated as n = 7 or n = 8 due to a typographical error. The correct sample size is n = 6 per group throughout, consistent with the Methods section and with the degrees of freedom reported in all statistical analyses (F (5,30)). The figure captions have been corrected accordingly to reflect n = 6 per group.

Second, an error occurred during the preparation of Figure 6. The representative micrograph in Panel B (NE group, CA1 region, H&E ×400) has been corrected. This correction does not affect the histological scoring, statistical analyses, or the conclusions of the study.

Corrected Figure 6:


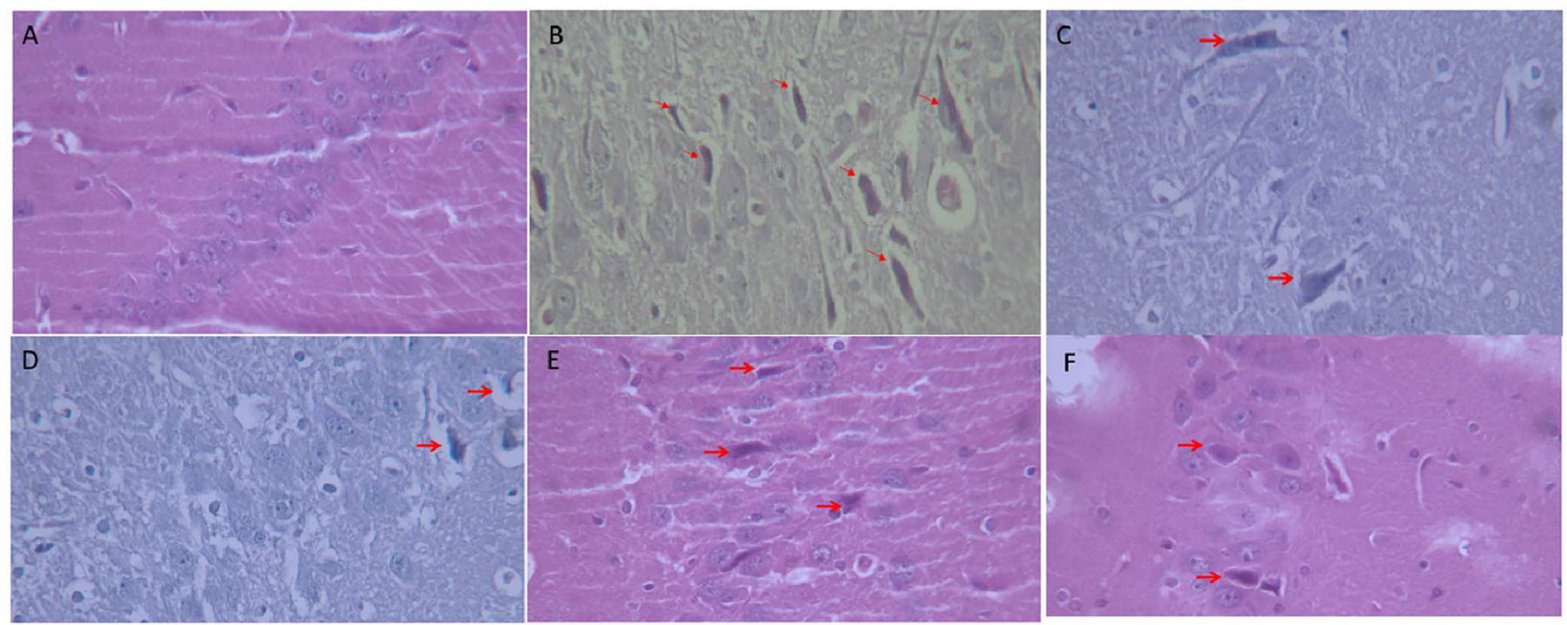


The authors apologize for these errors.

